# T Cell Phenotype and T Cell Receptor Repertoire in Patients with Major Depressive Disorder

**DOI:** 10.3389/fimmu.2018.00291

**Published:** 2018-02-20

**Authors:** Kostas Patas, Anne Willing, Cüneyt Demiralay, Jan Broder Engler, Andreea Lupu, Caren Ramien, Tobias Schäfer, Christian Gach, Laura Stumm, Kenneth Chan, Marissa Vignali, Petra C. Arck, Manuel A. Friese, Ole Pless, Klaus Wiedemann, Agorastos Agorastos, Stefan M. Gold

**Affiliations:** ^1^Institut für Neuroimmunologie und Multiple Sklerose (INIMS), Universitätsklinikum Hamburg-Eppendorf, Hamburg, Germany; ^2^Klinik für Psychiatrie und Psychotherapie, Universitätsklinikum Hamburg-Eppendorf, Hamburg, Germany; ^3^Immunomodulation Group, Cantacuzino National Research Institute, Bucharest, Romania; ^4^Fraunhofer IME ScreeningPort, Hamburg, Germany; ^5^Adaptive Biotechnologies, Seattle, WA, Unites States; ^6^Experimentelle Feto-Maternale Medizin, Universitätsklinikum Hamburg-Eppendorf, Hamburg, Germany; ^7^Charité – Universitätsmedizin Berlin, Humboldt Universität zu Berlin, Berlin Institute of Health (BIH), Klinik für Psychiatrie und Psychotherapie, Campus Benjamin Franklin (CBF), Berlin, Germany

**Keywords:** adaptive immunity, major depressive disorder, chemokine receptors, regulatory T cells, T cell receptor repertoire

## Abstract

While a link between inflammation and the development of neuropsychiatric disorders, including major depressive disorder (MDD) is supported by a growing body of evidence, little is known about the contribution of aberrant adaptive immunity in this context. Here, we conducted in-depth characterization of T cell phenotype and T cell receptor (TCR) repertoire in MDD. For this cross-sectional case–control study, we recruited antidepressant-free patients with MDD without any somatic or psychiatric comorbidities (*n* = 20), who were individually matched for sex, age, body mass index, and smoking status to a non-depressed control subject (*n* = 20). T cell phenotype and repertoire were interrogated using a combination of flow cytometry, gene expression analysis, and next generation sequencing. T cells from MDD patients showed significantly lower surface expression of the chemokine receptors CXCR3 and CCR6, which are known to be central to T cell differentiation and trafficking. In addition, we observed a shift within the CD4^+^ T cell compartment characterized by a higher frequency of CD4^+^CD25^high^CD127^low/−^ cells and higher *FOXP3* mRNA expression in purified CD4^+^ T cells obtained from patients with MDD. Finally, flow cytometry-based TCR Vβ repertoire analysis indicated a less diverse CD4^+^ T cell repertoire in MDD, which was corroborated by next generation sequencing of the TCR β chain CDR3 region. Overall, these results suggest that T cell phenotype and TCR utilization are skewed on several levels in patients with MDD. Our study identifies putative cellular and molecular signatures of dysregulated adaptive immunity and reinforces the notion that T cells are a pathophysiologically relevant cell population in this disorder.

## Introduction

Major depressive disorder (MDD) affects approximately 15% of adults over their lifespan (1) and is among the top two leading causes of years lived with disability worldwide (2). The etiopathogenesis of MDD is multifactorial and many neurobiological systems have been implicated (3, 4). Despite advanced understanding of the pathophysiology, approximately 30% of patients do not respond—even after several treatment attempts—to current antidepressants, which mainly target monoaminergic neurotransmission (5, 6). This underlines the need for approaches that target mechanisms beyond monoamine modulation (7).

Recently, accumulating experimental animal and human data have highlighted the importance of aberrant immunity in the development of depression (8). Similarly, epidemiological data have linked infections and autoimmunity to the risk of the disorder (9, 10). Exploring immune alterations in MDD may, thus, have the potential to open new avenues for treatment, since the immune system could be more amendable by therapeutic modulation than targets in the central nervous system (CNS).

Much of the research on immune dysfunction in MDD has focused on activation of the innate immune system (8). However, intriguing new evidence suggests that the different branches of the immune system might be differentially affected in MDD with relative impairment of adaptive immune responses (11), although this has not yet been explored in a targeted way on a cellular level. Moreover, preclinical studies have implicated the presence of a co-evolutionary link between T cell responses and CNS function (12, 13) and it is now increasingly recognized that the T cell compartment regulates cognition and mood-related behaviors in experimental animals (14, 15). Along similar lines, aberrant T cell differentiation and attenuated reactivity to CNS-derived antigens were hypothesized to play a role in MDD pathophysiology (8, 16). In view of these putative “antigen selection” pressures (i.e., infections and skewed specificity), we hypothesized that peripheral blood T cells from MDD patients would exhibit an altered phenotype as well as a biased profile in the T cell receptor (TCR) repertoire compared to non-depressed controls.

## Materials and Methods

### Subjects

We enrolled antidepressant-free patients with MDD but no other psychiatric comorbidities as well as non-depressed volunteers, matched pairwise for sex, age, current smoking status (yes/no), and BMI. All participants were of European descent.

*Inclusion criteria for MDD patients*: (a) psychiatrist-confirmed diagnosis of MDD, single or recurrent, according to DSM-IV criteria; (b) a minimum score of 18 points on the Hamilton Rating Scale for Depression (HRSD); (c) age 18–65 years; (d) at least 8 weeks free of any psychiatric medication (e.g., antidepressants, antipsychotics, and mood stabilizers).

*Inclusion criteria for non-depressed controls*: (a) no current or lifetime mood disorder diagnosis and (b) a score ≤5 on the Quick Inventory of Depressive Symptoms-Self Report (QIDS-SR).

*Exclusion criteria for all participants*: (a) past or present self-reported diagnosis of a major medical condition, including chronic or acute inflammatory, metabolic, and neurological disorders; (b) Axes I or II comorbidities; (c) regular use of either prescribed, over-the-counter medication or illicit drugs; thus, any subject on anti-inflammatory drugs, cholesterol-reducing drugs, and other possibly immune-modifying agents was excluded (see Table S1 in Supplementary Material); (d) drinking of more than 100 g of alcohol per week; (e) basic blood laboratory test values deviating significantly from the normal range; (f) current adverse life events (e.g., divorce, loss of job, and illness in the family); (g) pregnancy or nursing; and (h) recent vaccination (within the past month). Hypothyroidism in euthyroid state through hormonal substitution and hypertension in normotensive state through antihypertensive medication did not represent exclusion criteria (see Table S1 in Supplementary Material).

Patients were recruited through our specialized depression out-patient clinic program at the Department of Psychiatry and Psychotherapy, University Medical Center Hamburg-Eppendorf. Non-depressed controls were recruited from the same geographical region through advertisements and from the staff of the University Medical Center Hamburg-Eppendorf. All participants provided written informed consent before enrollment in the study. This study has been approved by the appropriate Ethics Review Committee (Ethik-Kommission der Ärztekammer Hamburg, Ethikvotum PV4161 and PV4719).

### Clinical Assessments

All subjects underwent detailed clinical assessments, including medical history, current medication, and psychiatric comorbidities. Diagnosis was established with the Structured Clinical Interview for the DSM-IV-TR Axis I Disorders (SCID-I) and depression severity was assessed using the HDRS by experienced board-certified psychiatrists (Cüneyt Demiralay and Agorastos Agorastos).

### Peripheral Blood Mononuclear Cells (PBMC) Isolation and Biobanking

We obtained approximately 30 ml of blood in S-Monovette K3 EDTA tubes and 7 ml in S-Monovette Serum-Gel tubes (Sarstedt). All samples were collected in the morning (8:00 a.m.). PBMCs were then isolated using a Ficoll–Hypaque gradient as described (17), aliquoted in RPMI containing 10% DMSO and 25% FCS at 1 × 10^7^ cells/ml, gradually cooled down to −80°C in a Mr. Frosty for 18 h and stored in liquid nitrogen until assayed. A small amount of whole blood (50 µl) was used for total and differential leukocyte counts using a Coulter Ac·T Diff hematology analyzer (Beckman Coulter). All other assays were performed using cryopreserved PBMCs or serum with one subject from each group run in parallel to control systematic variation in reagents used.

### Cell Purification

For qRT-PCR and TCR sequencing analyses, CD4^+^ T cells were purified using magnetic beads (negative selection by BD IMag Human CD4 T Lymphocyte Enrichment Set, BD Biosciences) from PBMC aliquots thawed in cell separation buffer (1% human serum, 2 mM EDTA in PBS). In our hands, this method typically yields a cell purity of approximately 95% for CD4^+^ T cells as confirmed by flow cytometry.

### Flow Cytometry

We used flow cytometry with hierarchical gating strategies adapted from established guidelines for analysis of human PBMCs and suitable for cryopreserved samples (18, 19). Our panel offered survey phenotyping of T cell subsets, including Tregs, as well as B cells and NK cells (see Figure S1 in Supplementary Material). The following fluorochrome-conjugated monoclonal antibodies were used: CD3 BV605 (OKT3, Biolegend), CD4 Alexa 700 (RPA-T4, Biolegend), CD8α V500 (SK1, BD Biosciences), CCR6 PE-Cy7 (G034E3, Biolegend), CXCR3 PE (G025H7, Biolegend), CD127 APC (A019D5, Biolegend), CD25 BV421 (M-A251, Biolegend), CD19 V500 (HIB19, BD Biosciences), CD56 PE-Cy7 (HCD56, Biolegend), CD20 PE (2H7, BD Biosciences), CD14 V450 (MFP9, BD Biosciences), and CD16 FITC (3G8, BD Biosciences). We also used the commercially available IOTest^®^ Beta Mark Kit (Beckman Coulter) for quantitative determination of the human TCR Vβ repertoire in CD4^+^ and CD8^+^ cells [nomenclature from Ref. (20)].

#### Surface Staining

To exclude dead cells from further analyses, we used the LIVE/DEAD Fixable Near-IR Dead Cell Stain Kit (Life Technologies) before applying any surface and intracellular stainings. Up to 1 × 10^6^ thawed PBMCs were washed with protein-free cold PBS (PAA Laboratories) at 485 g for 5 min at 4°C and then resuspended in 100 µl cold PBS containing the amine-reactive dye in 1:1,000 dilution. After light-protected incubation for 20 min at 4°C, cells were washed with cold PBS and subsequently stained for surface antigens, resuspended in 90 µl staining buffer (0.1% BSA, 0.02% NaN_3_ in PBS) and incubated with 0.1 µg/µl human IgG (Jackson ImmunoResearch) for 5 min at room temperature. Surface staining reactions were performed by light-protected incubation with 10 µl of Vβ-specific reagent mixture and/or 10 µl of 10× surface antibody cocktails for 30 min at 4°C. After washing with staining buffer at 485 g for 5 min at 4°C, cells were either resuspended in 250 µl staining buffer for acquisition or fixed for intracellular staining.

#### Intracellular Staining

Surface-stained cells were resuspended in 100 µl fixation buffer (Biolegend), incubated for 20 min at room temperature, washed twice with 1× permeabilization wash buffer (Biolegend) at 485 *g* for 5 min at 4°C and serially incubated with 0.1 µg/µl human IgG (5 min, at room temperature) and anti-CXCR3 antibodies for 30 min at room temperature. Cells were again washed twice with 1 ml permeabilization wash buffer and resuspended in 250 µl staining buffer for acquisition.

Data were acquired using a BD FACS LSR II flow cytometer and the FACS Diva v6.2 operating software. At least 1 × 10^5^ live lymphocytes were acquired from case–control samples during the same session and using the same acquisition settings. Variability of instrument performance was normalized by use of Cytometer Setup and Tracking beads (BD Biosciences). Data analysis and plotting were performed using FlowJo v10.0.8 (Tree Star).

### Serum Immunoassays for CXCL10 and CXCL11

CXCL10 and CXCL11 in sera were assayed with a multiplex bead-based immunoassay LEGENDplex (Biolegend) according to manufacturer’s instructions. For data acquisition and analysis, a BD FACS LSR II flow cytometer and the LEGENDplex v7.0 data analysis software were used, respectively.

### Serum Radioimmunoassays for ACTH and Cortisol

Stress hormone levels (ACTH and cortisol) were measured in sera obtained at 8:00 a.m. with commercially available radioimmunoassays (IBL IRMA and ICN Biomedicals RIA, respectively), according to manufacturer’s instructions.

### Reverse Transcription and Real-Time PCR

RNA was extracted from purified cell populations using RNeasy Plus Universal Mini Kit (Qiagen). 250–500 ng aliquots were used for cDNA synthesis by RevertAid H Minus First Strand cDNA Synthesis Kit (Thermo Scientific), followed by TaqMan assays (*FOXP3*: Hs01085834_m1, *T-bet*: Hs00203436_m1, *GATA3*: Hs00231122_m1, *RORC*: Hs01076122_m1) in ABI Prism 7900 HT Fast Real-Time PCR system (Applied Biosystems). All reactions were performed in triplicate. The expression levels of the genes of interest were calculated as 2^−ΔCt^ relative to the geometric mean expression of three housekeeping genes (*IPO8*: Hs00183533_m1, *TBP*: Hs00427620_m1, *RPL13A*: Hs04194366_g1), shown to be stably expressed in primary human T cells (21).

### Next Generation Sequencing of the TCRβ Repertoire

For sequencing of the CDR3 region in CD4^+^ T cell clones, we extracted total genomic DNA from negatively purified CD4^+^ T cells using the DNeasy Blood and Tissue Kit (Qiagen), according to manufacturer’s instructions. We then amplified and sequenced the CDR3 region of rearranged TCRβ genes, which were defined according to IMGT (22), using previously described immunosequencing protocols [Adaptive Biotechnologies (23)]. This provides an unbiased measurement of the frequencies of individual T cell clones. Raw sequence data were pre-processed by Adaptive Biotechnologies for PCR and sequencing error correction (23) and uploaded into the immunoSEQ^®^ Analyzer. Post-analysis was conducted using VDJtools v1.0.7 (24). Sequencing data are available using the following URL: http://doi.org/10.21417/B7RG6H.

### Statistical Analyses

All continuous variables are presented as median values with interquartile range, unless otherwise specified. Differences between patients and matched controls were tested for statistical significance using the paired Wilcoxon signed-rank test. For dichotomous variables, the McNemar’s test was used. Bivariate correlation analyses were conducted using Spearman’s rank correlation test. As a measure of Vβ repertoire skewing, we calculated the Gini-TCR index, as previously described (25). This index is a direct measure of TCR Vβ usage with higher values indicating higher clonality (i.e., less evenly distributed repertoire). All statistical analyses were performed using SPSS version 19 (IBM). A two-tailed *p* < 0.05 was considered significant and *p* < 0.10 was considered a trend. All graphs were made using GraphPad Prism v5.04, except for the pie charts in Figure 6, which were generated using the R package ggplot2.

## Results

### Descriptives

Clinical characteristics of the MDD and control groups can be found in Table 1. Patient level clinical characteristics including non-psychiatric medication are provided in Table S1 in Supplementary Material. Depression severity in the MDD group was moderate to severe. 18 MDD patients were antidepressant-naïve at the time of inclusion.

**Table 1:** Clinical characteristics.

	MDD (*n* = 20)	CTR (*n* = 20)	*p-*value*
Males/Females (*n*)	9/11	9/11	>0.99
Age (years), median (IQR)	36.5 (30.5–44)	37 (31–46)	0.28
BMI, median (IQR)	24.1 (20.7–27.8)	24.4 (22.8–27.1)	0.37
% Currently smoking (*n*)	35 (7)	35 (7)	>0.99
QIDS-SR, median (IQR)	18.5 (16.5–21)	2 (1–3.5)	<0.001
HRSD, median (IQR)	21.5 (19.25–24)	N/A	N/A

*MDD, major depressive disorder; CTR, non-depressed controls; BMI, body mass index; IQR, interquartile range; QIDS-SR, 16-item Quick Inventory of Depressive Symptoms-Self Report; HRSD, 17-item Hamilton Rating Scale for Depression, N/A, not applicable*.

*Based on the McNemar’s test for dichotomous variables and the Wilcoxon signed-rank test for continuous variables.

### Circulating Leukocyte Numbers and Frequencies

**Figure 1:**
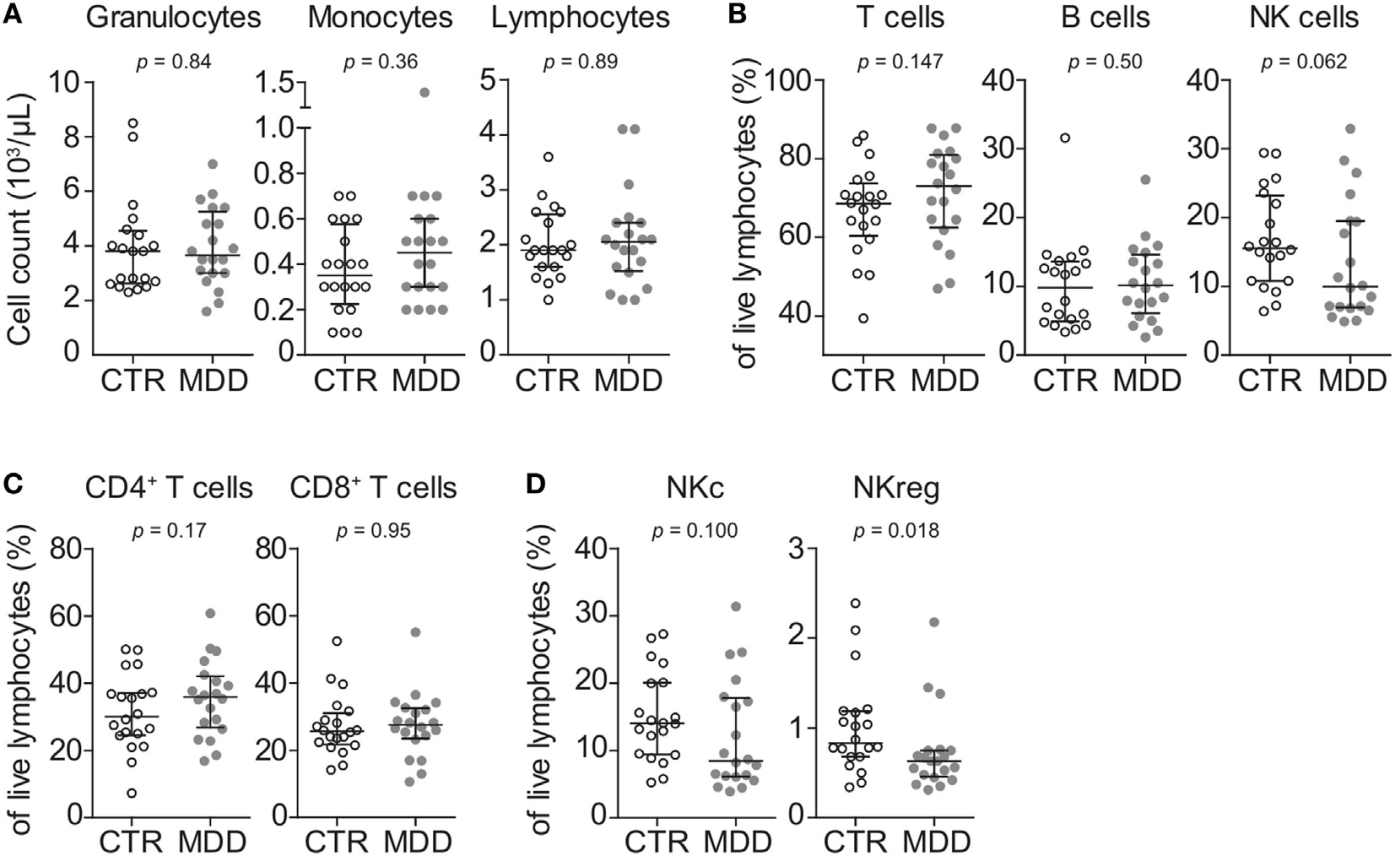
Peripheral blood counts and frequencies of major leukocyte subsets. **(A)** Absolute peripheral blood granulocyte, monocyte, and lymphocyte counts were obtained from major depressive disorder (MDD) patients and matched non-depressed controls (CTR) using a Coulter Ac·T Diff hematology analyzer (*n* = 40). **(B)** Frequencies of total CD3^+^ lymphocytes (T cells), CD3^−^CD56^−^CD19^+^ B cells and CD3^−^CD19^−^CD20^−^CD14^−^CD56^+^ natural killer (NK) cells were obtained by flow cytometric analysis of thawed peripheral blood mononuclear cells. **(C)** T cells were further discriminated into CD4^+^CD8^−^ and CD8^+^CD4^−^ subsets. **(D)** Among NK cells, CD56^low^CD16^+^ cytotoxic (NKc) cells and CD56^high^CD16^−^ regulatory (NKreg) cells were also identified. Graphs depict medians with interquartile ranges. For all comparisons, the Wilcoxon signed-rank test was used.

In order to obtain a general overview of immune cell composition in the peripheral blood, we first analyzed the absolute counts and frequencies of major leukocyte subsets. We observed no significant differences in absolute counts of circulating granulocytes, monocytes, and lymphocytes (Figure 1A), frequencies of major T cell subsets (CD4^+^ or CD8^+^) or B cells (Figures 1B,C). In line with recent studies (26–28), natural killer (NK) cells showed a trend toward lower frequency in MDD patients (*p* = 0.062; Figure 1B). Here, group differences were mainly driven by the putatively regulatory CD56^high^CD16^−^ NK subset (*p* = 0.018; Figure 1D).

### Chemokine Receptor Expression CXCR3 and CCR6

**Figure 2:**
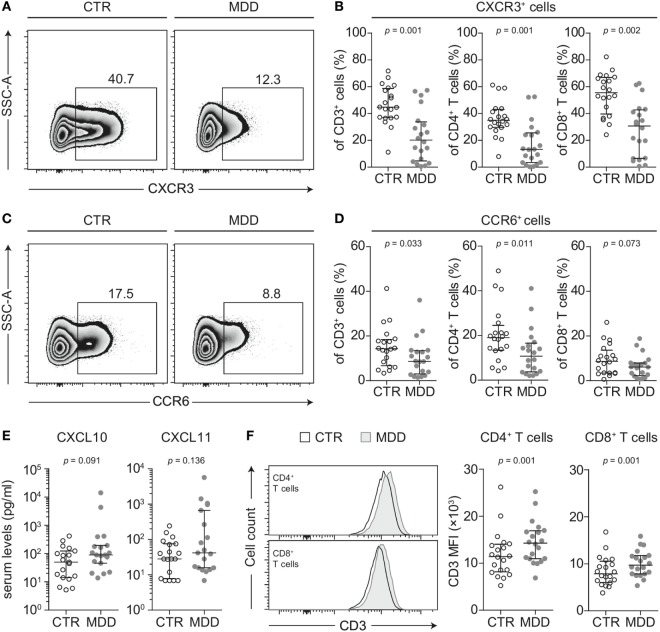
CXCR3 and CCR6 expression in T cells of major depressive disorder (MDD) patients and non-depressed controls. **(A)** CXCR3-expressing T cells were identified by flow cytometric analysis of peripheral blood mononuclear cells from MDD patients and matched non-depressed controls (CTR). Displayed values are frequencies of CXCR3^+^ T cells expressed as a percentage of live CD3^+^ lymphocytes from a representative case–control pair. **(B)** Percentages of CXCR3-expressing total T cells, CD4^+^, and CD8^+^ T cells were quantified in our cohort (*n* = 40). **(C,D)** Similar analyses were conducted for the surface expression of CCR6 on total T cells as well as on the CD4^+^ and CD8^+^ T cell subsets. **(E)** The CXCR3 ligands CXCL10 and CXCL11 were quantified in sera of MDD patients and matched controls using a cytometric bead array (*n* = 38). **(F)** Surface CD3 MFI levels were measured by flow cytometric analysis of CD4^+^ and CD8^+^ T cells from MDD patients and matched controls (*n* = 40). All graphs depict medians with interquartile ranges. For all comparisons, the Wilcoxon signed-rank test was used. SSC-A, side scatter-area; CXCR3, CXC-chemokine receptor type 3; CCR6, CC-chemokine receptor type 6; MFI, median fluorescence intensity.

To identify possible shifts in T cell phenotype, we next analyzed the expression of two chemokine receptors that are characteristic of effector T cell differentiation (CXCR3 and CCR6) (18). T cells of MDD patients showed significant reductions in the surface expression of both CXCR3 (*p* = 0.001; Figures 2A,B) and CCR6 (*p* = 0.033; Figures 2C,D). This was seen in both CD4^+^ and CD8^+^ T cell subsets for CXCR3 (Figure 2B) and mainly in CD4^+^ T cells for CCR6 (Figure 2D). In 17 out of our 20 case–control pairs, CXCR3 expression was lower in the MDD subjects.

**Figure 3:**
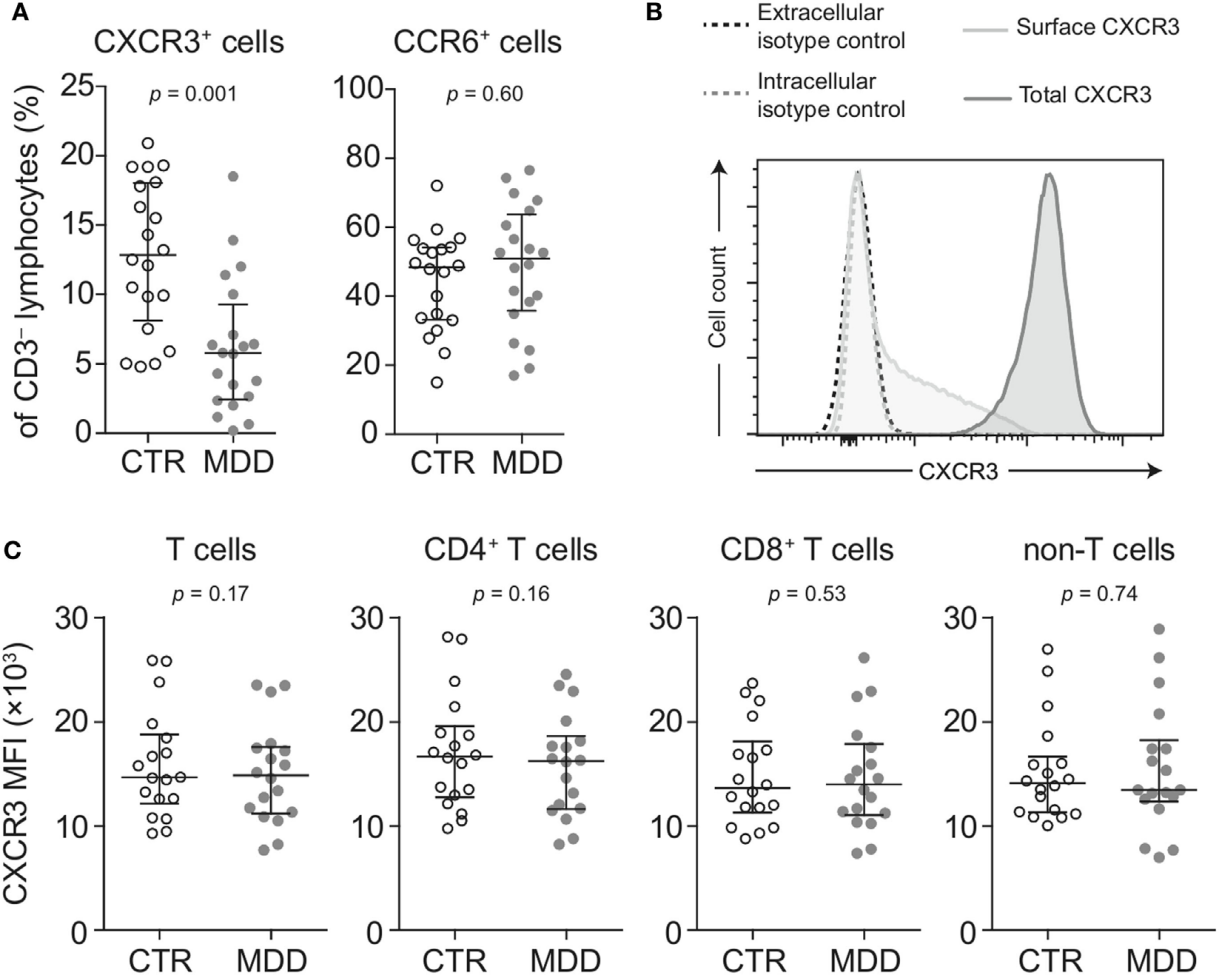
Surface and intracellular staining of CXCR3. **(A)** Percentages of CXCR3- and CCR6-expressing CD3^−^ lymphocytes (non-T cells) were quantified in major depressive disorder (MDD) patients and matched non-depressed controls (CTR) (*n* = 40). **(B)** A representative plot shows fluorescence intensity of CXCR3 expression in intact (surface CXCR3; light gray-shaded curve) relative to fixed and permeabilized T cells (total cellular CXCR3; dark gray-shaded curve). Isotype-matched negative controls were used at the same concentration before fixation (black-dashed curve) and after fixation-permeabilization (gray-dashed curve) and showed no positive staining for CXCR3. **(C)** Total cellular CXCR3 MFI levels were measured by flow cytometric analysis of fixed and permeabilized peripheral blood mononuclear cells (PBMCs) from MDD patients and matched controls (*n* = 36). Stained PBMCs were gated on live CD3^+^ lymphocytes (T cells), CD4^+^ and CD8^+^ T cell subsets as well as CD3^−^ lymphocytes (non-T cells). Graphs depict medians with interquartile ranges. For all comparisons, the Wilcoxon signed-rank test was used. CXCR3: CXC-chemokine receptor type 3; CCR6: CC-chemokine receptor type 6; MFI: median fluorescence intensity.

Lower surface CXCR3 expression was not T cell-specific, as the percentage of CXCR3-expressing CD3^−^ lymphocytes (non-T cells) was also found to be significantly lower in MDD patients (*p* = 0.001; Figure 3A). However, lower surface expression of CCR6 was confined to T cells, as we detected no difference in the frequency of CCR6-expressing CD3^−^ lymphocytes between patients and controls (*p* = 0.60; Figure 3A).

Next, we conducted several analyses to explore potential mechanisms that could underlie the extensive loss of surface CXCR3 expression in MDD. We found no differences in total cellular levels of CXCR3 (i.e., surface and intracellular combined) in T cells and non-T cells (Figures 3B,C), suggesting different receptor turnover at the cell membrane in MDD.

T cells rapidly internalize CXCR3 when exposed to CXCL10 and CXCL11 (29) and there is evidence to suggest that CXCL10 is elevated in MDD (30). In our sample, serum CXCL10 tended to be higher in MDD, although this difference reached only a trend (*p* = 0.091; Figure 2E). Descriptively, CXCL11 was also elevated but this did not reach statistical significance (Figure 2E). However, the intercorrelation between the two chemokines was robust (Spearman’s rho = 0.862, *p* < 0.001).

CD3 stimulation can be an alternative mechanism accounting for downregulation of both CXCR3 and CCR6 from the cell surface (31). In line with this hypothesis, we observed higher median fluorescence intensity of CD3 expression in T cells of MDD patients (*p* = 0.001; Figure 2F). It has been previously described that upregulation of surface CD3 is associated with prolonged TCR engagement (32), which provided further rationale for our hypothesis that MDD patients may bear TCR repertoires of biased clonal composition (see T cell repertoire analyses).

### CD4^+^ T Cell Subsets and Transcription of Master Regulators of T Cell Differentiation

**Figure 4:**
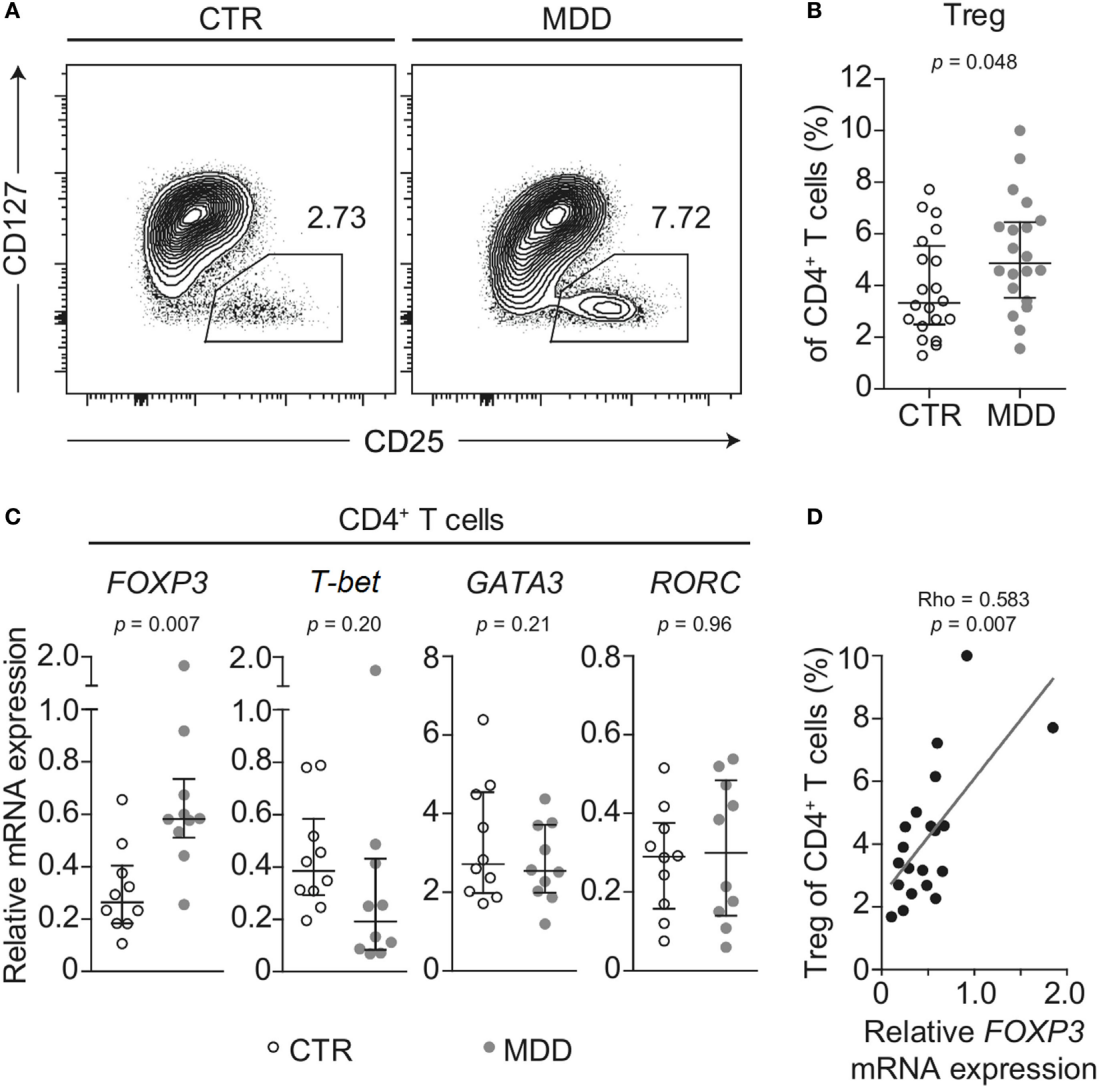
Regulatory T cells in major depressive disorder (MDD) patients and non-depressed controls. **(A)** Regulatory T cells (Tregs) were identified by flow cytometric analysis of peripheral blood mononuclear cells from MDD patients and matched non-depressed controls (CTR). Displayed values are frequencies of Tregs expressed as a percentage of live CD4^+^ T cells from a representative case–control pair. **(B)** Differences in Treg frequency are depicted for the entire cohort (*n* = 40). **(C)** Negatively selected CD4^+^ T cells from a subsample of patients and matched controls (*n* = 20) were analyzed for mRNA expression of the T helper-associated transcription factors Forkhead box P3 (*FOXP3*), T-box 21 (*T-bet*), GATA binding protein 3 (*GATA3*), and RAR related orphan receptor C (*RORC*), respectively. Expression was normalized to the geometric mean expression of three housekeeping genes (*IPO8, TBP, RPL13A*). **(D)** The correlation between the expression levels of the gene *FOXP3* in purified CD4^+^ T cells and the frequency of Tregs expressed as a percentage of CD4^+^ T cells is plotted (*n* = 20). Graphs depict medians with interquartile ranges. For all comparisons, the Wilcoxon signed-rank test was used.

Shifts in CD4^+^ T cell subsets could indicate another aspect of skewed T cell differentiation in MDD (16). Indeed, we observed that MDD patients exhibited significantly higher frequency of CD25^high^CD127^low/−^ T cells (*p* = 0.048; Figures 4A,B), a surface phenotype associated with regulatory T (Treg) cells in humans (19, 33). In addition, in a subset of 10 case–control pairs with sufficient biomaterial available, we observed increased mRNA levels of the Treg transcription factor *FOXP3* (*p* = 0.007), while no significant changes were observed in the levels of mRNA for transcription factors linked to differentiation of the Th1 (*T-bet*), Th2 (*GATA3*), or Th17 (*RORC*) lineages (Figure 4C). Corroborating a relative shift toward a Treg-associated phenotype at the expense of Th1 differentiation, *FOXP3* mRNA expression was significantly and positively correlated with CD25^high^CD127^low/−^ frequency (Spearman’s rho = 0.583, *p* = 0.007; Figure 4D) but negatively correlated with the frequency of CXCR3-expressing CD4^+^ T cells (Spearman’s rho = − 0.555, *p* = 0.011). No correlation was found for CCR6-expressing CD4^+^ T cells (Spearman’s rho = −0.194, *p* = 0.41).

### T Cell Repertoire Analysis

**Figure 5:**
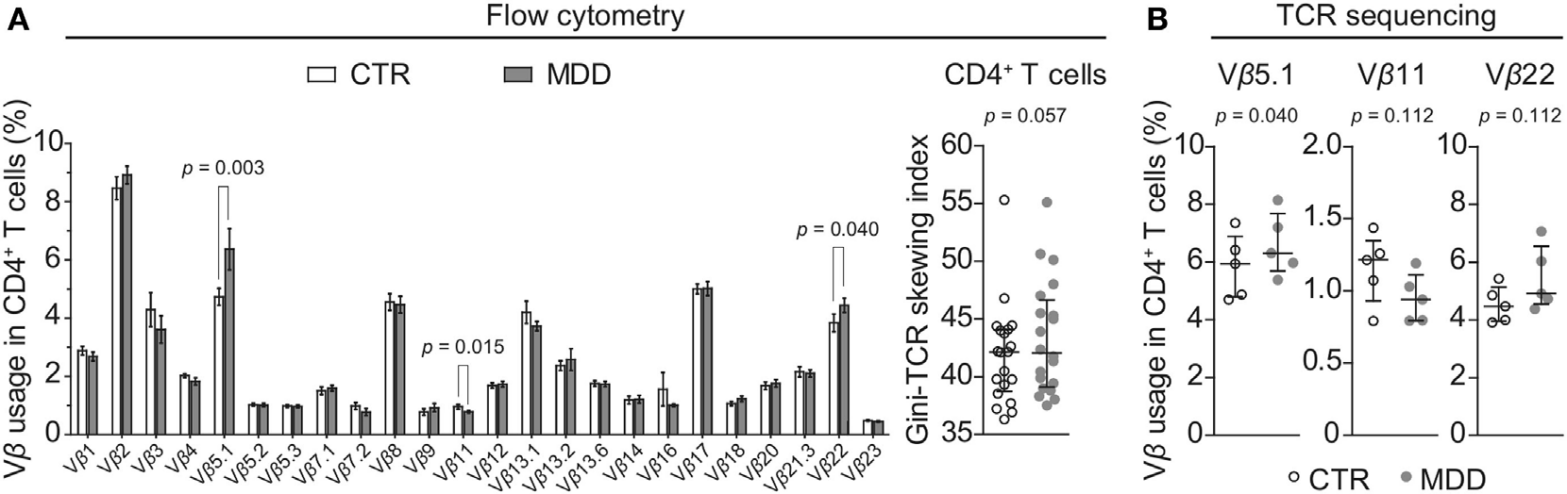
CD4^+^ T cell repertoire in major depressive disorder (MDD) patients and non-depressed controls. **(A)** T cell receptor (TCR) variable β chain (Vβ) family distribution analysis was performed by means of flow cytometric interrogation of CD4^+^ T cells from MDD patients and matched non-depressed controls (CTR) (*n* = 40). The resulting clonogram represents percentages (mean ± SEM) of the usage of 24 Vβ families. The Gini-TCR skewing index was next applied to the flow cytometric Vβ repertoire analysis (graph on right). Significant *post hoc* comparisons are denoted for the families Vβ 5.1, Vβ 11, and Vβ 22 (two-tailed, uncorrected *p*-values). **(B)** CD4^+^ T cells were negatively selected from a subsample of patients and matched controls (*n* = 10) and total genomic DNA was extracted for next generation sequencing of the TCRβ CDR3 repertoire. Usage of the families Vβ 5.1 (TCRBV05-01), Vβ 11 (TCRBV25-01), and Vβ 22 (TCRBV02-01) was then followed up (one-tailed planned comparisons). For all comparisons, the Wilcoxon signed-rank test was used.

In order to explore whether a skewed T cell phenotype in MDD is paralleled by a biased TCR expression profile, we examined TCR Vβ family distribution by flow cytometry-based analysis of the entire cohort (*n* = 40). Overall, the T cell repertoire of MDD patients showed a trend for higher Gini-TCR indices in CD4^+^ cells (*p* = 0.057; Figure 5A), indicating that the CD4^+^ TCR repertoire might be less evenly distributed in MDD. By contrast, no evidence for skewing of the CD8^+^ T cell repertoire was observed (*p* = 0.36; data not shown).

**Figure 6:**
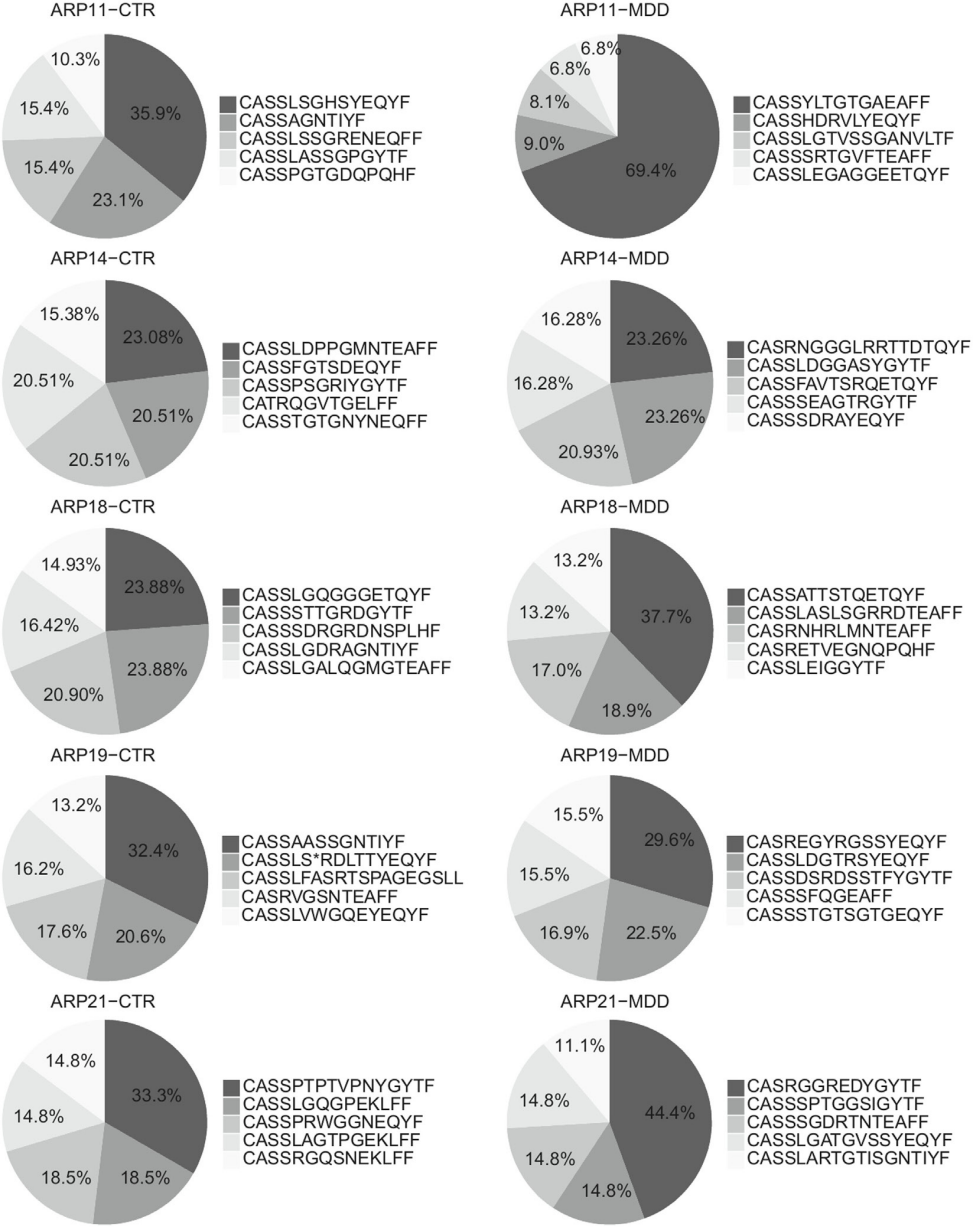
Top Vβ 5.1-expressing clones. Relative frequencies of top five clones expressing Vβ 5.1 (TCRBV05-01) and their CDR3 amino acid sequence in patients with major depressive disorder (MDD) and matched non-depressed controls (CTR) (*n* = 10).

Among CD4^+^ T cells, we found significantly higher usage of the Vβ families 5.1 and 22 (unadjusted *p* = 0.003 and *p* = 0.040, respectively; Figure 5A) and lower usage of Vβ 11 (unadjusted *p* = 0.015; Figure 5A). Next, we sought to confirm these observations by using next-generation sequencing in a subgroup of five case–control pairs, which were chosen in a manner blind to chemokine receptor expression and Gini-TCR index differences (see Table S1 in Supplementary Material). Sequencing of the beta chain CDR3 region in purified CD4^+^ T cells confirmed the increased usage of Vβ 5.1 (TCRBV05-01) in the MDD group as compared to the control group (*p* = 0.040, one-tailed planned comparison; Figure 5B). Differences regarding Vβ 11 (TCRBV25-01) and Vβ 22 (TCRBV02-01) showed the same pattern as the flow cytometry analysis in the whole cohort but did not reach statistical significance (Figure 5B). The top five Vβ 5.1 T cell clones for each subject, which were all “private” clones, are displayed in Figure 6.

### Association between Immune and Neuroendocrine Variables

In order to explore whether T cell alterations are associated with neuroendocrine dysregulation in MDD patients (34), we conducted correlation analyses between cellular immune measures found to differ between the MDD and control groups and serum levels of stress hormones (ACTH and cortisol). We found no associations of ACTH or cortisol levels with our T cell phenotype and TCR repertoire outcomes (all *p* > 0.15, data not shown).

## Discussion

Here, we provide converging evidence from several cellular and molecular approaches that antidepressant-free MDD patients display biased T cell phenotype and TCR repertoire as compared to closely matched, non-depressed controls. Peripheral blood T cells from MDD patients showed a shift in the CD4^+^ T compartment toward Treg cells, paralleled by lower surface expression of the T helper differentiation-related chemokine receptors CXCR3 and CCR6. In addition, a less diverse TCR utilization profile was seen within the CD4^+^ but not the CD8^+^ T cell subset.

Our cellular immune markers did not show any significant associations with circulating stress hormone levels (ACTH, cortisol); therefore, changes in T cell phenotype and TCR repertoire are unlikely to simply represent an epiphenomenon of neuroendocrine dysregulation in MDD (34). Furthermore, given the range of group differences in surface chemokine receptor expression as well as shifts in CD4^+^ T cell phenotype, our observations probably reflect a skewing in T cell differentiation, rather than implicating single molecules in MDD pathophysiology. Nevertheless, it is worthwhile to consider the potential functional implications of some of the candidate markers we identify in this study.

CXCR3 is a chemokine receptor that is highly expressed on effector CD4^+^ and CD8^+^ T cells and grants them entry into otherwise restricted sites of Th1-type inflammation and infection (35). Although we have not tested the functional consequences of lower surface CXCR3 in MDD directly, it has been previously shown that ligand-induced internalization of this receptor is associated with abolished migratory capacity of both CD4^+^ and CD8^+^ human T cells toward a cognate ligand (36). Furthermore, animal studies have shown that CXCR3 enhances the ability of T cells to safeguard against infections (37–39). Whether or not T cells from patients with MDD have a functional impairment with regard to their ability to respond to and clear infections should be, thus, investigated in future studies.

Intriguingly, animal models have shown that virus-induced “sickness-behavior,” which bears symptomatic and immunological similarities to MDD (40, 41), depends on the CXCL10-CXCR3 axis (42). Therefore, lower expression of CXCR3 as reported here might directly be involved in MDD pathophysiology. Along similar lines, a post mortem study showed that CXCR3^+^ T cells can be found in the human CNS, hinting at a potential role of these cells for maintenance and/or immune surveillance in the brain (43). Whether or not lower surface expression of CXCR3 in peripheral blood T cells might interfere with these functions, however, remains speculative. Ideally, this should further be explored in appropriate animal models, where immune cell trafficking into the CNS as well as peripheral tissues can be monitored.

Another functional implication of our findings could be overly suppressed host T cell responses in MDD owing to a bias toward Treg differentiation. It is worth noting that similar phenomena have been observed in chronic viral infections (44) as well as in animal models of neuropsychiatric disorders (45, 46).

Strengths of our study include the detailed clinical characterization and homogeneity of our patient cohort with moderate to severe MDD but no other psychiatric diagnoses or major somatic comorbidities. Further minimizing potential confounds, our study used close pairwise matching of cases and controls for variables that likely have an impact on immune function, such as sex, age, BMI, and smoking status. Moreover, all participants were non/mild drinkers and we only included currently unmedicated and mostly antidepressant-naïve MDD patients, using a clear cut-off in depression severity (HDRS score of 18 or more).

However, some limitations of our study have to be considered. First, no data were available for other clinical variables that may have influenced immune function, e.g., physical activity and nutrition (47). All MDD subjects were untreated with antidepressants and only four participants (*n* = 2 in the MDD group and *n* = 2 in the control group) were receiving non-psychiatric medications that were allowed in the study. Having said that, it is not possible to assess and control all potential additional factors that might be associated with MDD in cross-sectional case–control studies in humans. Thus, future longitudinal studies will be required to better understand the factors linked to the immune abnormalities observed herein. Second, the strict matching and the additional exclusion criteria applied increased the clinical homogeneity of the MDD group, but also led to a comparatively small study sample. This obviously has implications for the generalizability of our findings.

Beyond the advantages of more homogeneous patient groups in research studies, even in this small sample, we quite closely replicated previous immunological findings in MDD [e.g., elevated serum levels of CXCL10 (30)]. Furthermore, our results are in line with a recent whole blood transcriptomic analysis, which identified lower *CCR6* transcripts in both antidepressant-treated and antidepressant-free MDD cohorts (48) and our findings on lower NK cell frequency are consistent with lower expression of NK-related genes in MDD (26). Thus, we are confident that our well-characterized cohort is representative of MDD patients.

Our results on higher Treg frequency are consistent with recent reports showing a higher percentage of CD25^+^CD127^low^CCR4^+^ Tregs in antidepressant-free depressed patients (28) and a positive association between the frequency of CD25^high^CD127^low^ Tregs and depressive symptoms in older adults following an acute stressor (49). However, our results are in conflict with other previous studies indicating lower frequency of Tregs in MDD patients (27, 50). One possible explanation for this discrepancy could be differences in the clinical characteristics of the study samples (medication status, age, BMI). In addition, methodological differences in Treg definition could also have contributed to these discrepancies so that functional analyses of Treg suppressive capacity will be needed in the future to more specifically determine the role of Tregs in MDD.

In summary, we provide converging evidence from molecular and cellular analyses for a skewed T cell phenotype and CD4^+^ T cell repertoire in antidepressant-free MDD patients. These findings from our hypothesis-driven study should be confirmed in larger studies and expanded by employing unbiased systems biology approaches. It is important to note that besides MDD, other psychiatric disorders such as schizophrenia have been linked to immune alterations. In schizophrenia, many of the known risk genes are involved in immune regulation (51) and data from animal models, clinical studies, and epidemiology support a role of the immune system in its pathobiology (c.f. (52) for a recent review). Moreover, meta-analyses have confirmed changes in lymphocyte subset counts and frequencies (53) and cytokine levels (54). However, it should be noted that both on a genetic level (55) as well as with regard to immunological parameters such as cytokine levels (56), there is considerable overlap between major psychiatric disorders, including MDD, schizophrenia, and bipolar disorder. Thus, more work is needed to determine if any immune markers are specific to a given disorder or maybe linked to a specific symptom domain observed across diagnostic categories. Ultimately, this may have the potential to open new avenues for research toward an immunotherapy for MDD.

## Ethics Statement

This study has been approved by the appropriate Ethics Review Committee (Ethik-Kommission der Ärztekammer Hamburg, Ethikvotum PV4161 and PV4719). All participants provided written informed consent before enrolment in the study.

## Author Contributions

Conception and design: KP and SMG. Execution of experiments: KP, AL, CR, TS, CG, KC, and MV. Acquisition of data: KP., CD, LS, and AA. Analysis of data: KP, AW, JE, and AA. Interpretation of data: KP, AW, PA, MF, OP, KW, AA, and SMG. Obtained funding: AA, OP, and SMG. Drafting of the manuscript: KP and SMG. Revision of the manuscript for important intellectual content: AW, CD, JE, AL, CR, TS, CG, LS, KC, MV, PA, MF, OP, KW, and AA.

## Conflict of Interest Statement

KP, AW, CD, JE, AL, CR, CG, TS, LS, and KW have no potential conflicts of interest to disclose. PA has received research funding from the Deutsche Forschungsgemeinschaft (DFG). MF has received research funding from the Deutsche Forschungsgemeinschaft (DFG) and the Federal Ministry of Education and Research (BMBF). OP has received research funding from the Federal Ministry of Education and Research (BMBF). AA has received research funding from the Werner Otto Foundation. SG has received honoraria from Mylan GmbH and research funding from the Deutsche Forschungsgemeinschaft (DFG), the Federal Ministry of Education and Research (BMBF), the National MS Society (NMSS), the Werner Otto Foundation, and the European Commission. KC and MV have employment and equity ownership with Adaptive Biotechnologies.
