# Supplementary material for: T Cell Phenotype and T Cell Receptor Repertoire in Patients with Major Depressive Disorder
Source: Front Immunol. 2018 Feb 20;9:291. doi: 10.3389/fimmu.2018.00291 (PMC5826233; doi:10.3389/fimmu.2018.00291)
Supplement: Supplementary file 1 [file Data_Sheet_1.PDF]

Supplementary Material

"T cell phenotype and T cell receptor repertoire in patients with major depressive disorder"

by Patas *et al.*

Table S1.
Clinical characteristics of MDD patients and non-depressed controls, matched for age, sex, body mass index and smoking status (n = 40).

Figure S1.
Gating strategy.

Table S1: Clinical characteristics of MDD patients and non-depressed controls, matched for age, sex, body mass index and smoking status (*n* = 40)

Case Control	Age	Sex	BMI	Smoking	Previous episodes	QIDS-SR	HRSD	Medication	TCR Seq
ARP2-MDD	29	M	25.95	No	0	15	20		
ARP2-CTR	27	M	25.53	No	N/A	1	N/A		
ARP3-MDD	41	F	17.93	Yes	0	18	22		
ARP3-CTR	41	F	17.01	Yes	N/A	4	N/A		
ARP4-MDD	32	M	24.17	No	1	17	20		
ARP4-CTR	31	M	22.86	No	N/A	1	N/A		
ARP5-MDD	49	M	23.99	No	0	21	23	Ramipril	
ARP5-CTR	47	M	25.99	No	N/A	5	N/A		
ARP6-MDD	24	M	24.11	No	1	19	24		
ARP6-CTR	24	M	24.81	No	N/A	3	N/A		
ARP7-MDD	20	M	19.75	Yes	1	9	21		
ARP7-CTR	21	M	21.06	Yes	N/A	2	N/A		
ARP8-MDD	52	F	20.70	Yes	1	17	30		
ARP8-CTR	53	F	25.47	Yes	N/A	4	N/A		
ARP9-MDD	53	F	20.83	Yes	1	21	18		
ARP9-CTR	55	F	20.20	Yes	N/A	2	N/A	Bisoprolol, Potassium iodide	
ARP10-MDD	42	F	32.87	No	1	15	20	Ramipril	
ARP10-CTR	49	F	30.71	No	N/A	1	N/A		
ARP11-MDD	34	F	30.85	No	3	14	20		X
ARP11-CTR	32	F	33.06	No	N/A	1	N/A	Oral contraception	X
ARP12-MDD	27	F	26.26	No	0	23	19		
ARP12-CTR	30	F	27.40	No	N/A	5	N/A		
ARP13-MDD	30	F	23.33	No	1	16	19		
ARP13-CTR	33	F	23.05	No	N/A	4	N/A	L-Thyroxine	
ARP14-MDD	41.5	F	22.49	No	0	19	18		X
ARP14-CTR	42	F	22.86	No	N/A	1	N/A		X
ARP15-MDD	37	M	20.67	Yes	2	17	23	Zopiclone	
ARP15-CTR	43	M	22.83	Yes	N/A	0	N/A		
ARP16-MDD	61	M	28.07	No	0	20	22		
ARP16-CTR	65	M	24.16	No	N/A	1	N/A		
ARP17-MDD	36	F	26.23	No	1	23	28		
ARP17-CTR	35	F	24.09	No	N/A	3	N/A		
ARP18-MDD	32	F	17.58	Yes	3	19	24		X
ARP18-CTR	36	F	22.49	Yes	N/A	0	N/A		X
ARP19-MDD	35	F	32.79	No	3	18	19		X
ARP19-CTR	33	F	39.06	No	N/A	1	N/A		X
ARP20-MDD	46	M	28.03	Yes	0	26	24		
ARP20-CTR	41	M	24.57	Yes	N/A	3	N/A		
ARP21-MDD	38.5	M	27.16	No	0	24	26		X
ARP21-CTR	38	M	28.63	No	N/A	3	N/A		X

MDD: major depressive disorder; CTR: non-depressed control; BMI: body mass index; QIDS-SR: 16-item Quick Inventory of Depressive Symptoms-Self Report; HRSD: 17-item Hamilton Rating Scale for Depression; TCRseq: T cell receptor sequencing; N/A: not applicable

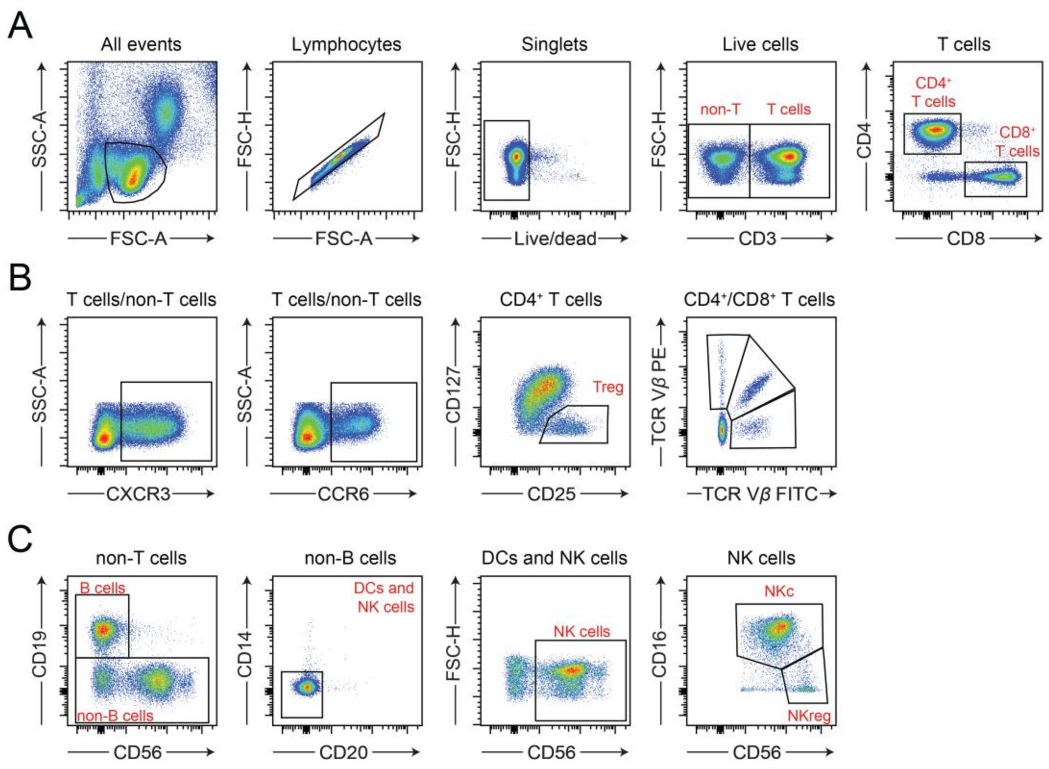

Figure S1: Gating strategy. (**A**) A common gating was applied across panels allowing for the identification of lymphocytes by forward (FSC) and side scatter (SSC), followed by exclusion of doublets and dead cells. Singlet live lymphocytes were selected on either CD3 positivity (T cells) or negativity (non-T cells). T cells were discriminated into $CD4^{+}CD8^{-}$ and $CD8^{+}CD4^{-}$ subsets. (**B**) T cells and non-T cells were further distinguished based on surface expression of CXCR3 and CCR6. Within $CD4^{+}$ T cells, regulatory T cells (Treg) were identified as $CD127^{low/-}$ while expressing high levels of CD25. The distribution of 24 Vβ families (3 Vβ specificities per tube) was assessed among $CD8^{+}$ and $CD4^{+}$ T cells. (**C**) Among non-T cells, B cells were defined as $CD56^{-}CD19^{+}$ and natural killer (NK) cells as $CD19^{-}CD20^{-}CD14^{-}CD56^{+}$. Within NK cells, $CD56^{low}CD16^{+}$ cytotoxic (NKc) and $CD56^{high}CD16^{-}$ regulatory (NKreg) subsets were identified as well. CXCR3: CXC-chemokine receptor type 3; CCR6: CC-chemokine receptor type 6; TCR: T cell receptor; DCs: dendritic cells
